# Influence of Fetal and Maternal Genetic Susceptibility to Obesity on Birthweight in African Ancestry Populations

**DOI:** 10.3389/fgene.2018.00511

**Published:** 2018-11-02

**Authors:** Deepika Shrestha, Mohammad L. Rahman, Tsegaselassie Workalemahu, Chunming Zhu, Fasil Tekola-Ayele

**Affiliations:** ^1^Epidemiology Branch, Division of Intramural Population Health Research, Eunice Kennedy Shriver National Institute of Child Health and Human Development, National Institutes of Health, Bethesda, MD, United States; ^2^Division of Intramural Population Health Research, Eunice Kennedy Shriver National Institute of Child Health and Human Development, National Institutes of Health, Bethesda, MD, United States

**Keywords:** obesity, birthweight, pregnancy, genetic risk score, African ancestry

## Abstract

Fetal and maternal genetic propensity to obesity can influence birthweight. We investigated the effects of fetal and maternal genetic risk of obesity on birthweight and evaluated whether these genetic influences modify the well-known association between maternal pre-pregnancy body mass index (BMI) and birthweight. In 950 mother-baby pairs of African ancestry, a genetic risk score for adulthood obesity was generated for mothers (mGRS) and their babies (bGRS) as the weighted sum of BMI-increasing alleles of 97 single nucleotide polymorphisms known to be associated with BMI. The median GRS value was used as a cut-off to define high or low bGRS and mGRS. High bGRS was significantly associated with 70 g lower birthweight (95% Confidence Interval [CI] = −127.4 to −12.4) compared to low bGRS. mGRS was positively correlated with birthweight but the association was not significant. mGRS modified the significant birthweight-increasing effect of maternal pre-pregnancy BMI (*P-for-interaction* = 0.03); among mothers with low mGRS, those who were overweight or obese had 127.7 g heavier babies (95% CI = 27.1 to 228.2) compared to those who had normal weight. In summary, fetal obesity genetic risk loci exert direct influence on birthweight, and maternal loci modify the effect of pre-pregnancy BMI on birthweight.

## Introduction

Maternal pre-pregnancy obesity, offspring birthweight, and offspring risk of obesity in later life are interlinked by interplays between genetic and environmental factors (Liu et al., 2016). Importantly, offspring birthweight is influenced by both fetal and maternal genes (Horikoshi et al., 2013, 2016; Beaumont et al., 2018). Recent genome-wide association studies (GWAS) in predominantly European ancestry populations have discovered 60 fetal and 10 maternal genetic loci associated with birthweight (Horikoshi et al., 2013, 2016; Beaumont et al., 2018). Genome-wide autosomal single nucleotide polymorphisms (SNPs) from fetal and maternal genotypes explained 15 and 11% of the variance in birthweight, respectively (Horikoshi et al., 2016; Beaumont et al., 2018). Fetal genetic loci associated with birthweight are enriched in several biological processes such as insulin signaling and cholesterol biosynthesis involved in metabolism (Horikoshi et al., 2016), and most maternal genes implicated in birthweight appear to exert their effects via regulation of maternal intrauterine glycemia (Beaumont et al., 2018). However, to date, the link between the fetal and maternal birthweight GWAS loci and obesity is not clearly understood.

Higher maternal pre-pregnancy body mass index (ppBMI) is also associated with increased birthweight (Liu et al., 2016). Maternal genetic loci associated with BMI may influence birthweight by modulating maternal obesity status or the intrauterine environment. The prevalence of maternal pre-pregnancy obesity increased by 8% in the United States between 2011–2015 and continues to rise globally (Deputy et al., 2018). Cumulating evidence shows that the rising prevalence of complex diseases such as obesity is due to complex gene-environment interactions, particularly interactions between risk genetic variants and environmental factors such as diet (Perusse and Bouchard, 2000), food preference (Bauer et al., 2009), and physical activity (Reddon et al., 2016). Moreover, the effect of genetic risk on BMI has been found to be higher among people exposed to obesity-precipitating environments and among African ancestry than European ancestry populations (Walter et al., 2016). Additionally, the incidence of low birthweight is considerably higher among African than European ancestry populations even after adjusting for socio-economic attributes (Foster et al., 1993; Collins et al., 2004). Therefore, detailed understanding of the relationships between maternal obesity genetic risk and birthweight in African ancestry populations is valuable, but still lacking.

Large-scale GWAS findings highlight strong genetic correlations between birthweight and obesity in adulthood (Horikoshi et al., 2016), suggesting that shared genetics likely explains part of the association between birthweight and obesity risk in later life (Barker et al., 1992). Previous studies have found that the effect of genes associated with BMI in adults begins in early childhood (Warrington et al., 2013). However, there is paucity of data on the extent to which maternal and fetal obesity genetic risk is linked to offspring birthweight, particularly in African ancestry populations, in which the rate of low birthweight is disproportionately high in the United States and globally (Martin et al., 2015; Kiserud et al., 2017).

The number of genetic loci robustly associated with obesity through GWAS is growing (Locke et al., 2015), and is providing an opportunity to improve our understanding of the genetic links between obesity and fetal growth. Studies that investigated the association between the burden of obesity genetic risk determined by a genetic risk score (GRS) and birthweight reported inconsistent findings (Andersson et al., 2010; Elks et al., 2010, 2012b, 2014; Kilpelaeinen et al., 2011; Belsky et al., 2012; Warrington et al., 2013; Li et al., 2017). A major limitation of these studies was that the number of SNPs forming the GRS were much smaller than the total number of presently known obesity genetic risk loci. In addition, the studies did not investigate the effect of fetal genetic risk on birthweight that is independent of the effect of maternal genetic risk on birthweight and vice-versa. None of these studies were performed in African ancestry populations, limiting generalizability of the findings.

The present study was performed to test for associations between genetic susceptibility to adulthood obesity and birthweight using genome-wide SNPs data from an African ancestry population collected in the Hyperglycemia Adverse Pregnancy Outcome (HAPO) study. A total of 97 SNPs associated with BMI in large-scale GWAS (Locke et al., 2015) were used to create GRS. We investigated the associations of fetal and maternal genetic susceptibility to adulthood obesity and birthweight and evaluated whether these genetic influences modify the well-known association between ppBMI and birthweight.

## Materials and Methods

### Study Cohort and Setting

We utilized genotype and phenotype data from the HAPO study (2001–2006). The HAPO study was an observational study that enrolled over 25,000 pregnant women from nine countries designed to examine the association between gestational hyperglycemia and newborn outcomes. Study methods of the HAPO study have been published previously (Hapo Study Cooperative Research Group et al., 2008). Informed written consent was obtained from all participants, and study protocols were approved by the local regional or institutional ethics committees. This study was conducted in accordance with the principles expressed in the Declaration of Helsinki.

For the present study, we included 1,250 Afro-Caribbean (Barbados) mother and offspring who had genotype data. The proportion of African genetic ancestry in the samples was determined using a model-based estimation of ancestry implemented in the program ADMIXTURE (Alexander et al., 2009). After excluding 300 participants who had missing data on obesity genetic risk genotypes, birthweight, and those for whom the proportion of African ancestry was less than 10%, 950 mother-baby pairs were taken forward for analysis.

Birthweight was measured using a calibrated electronic scale by trained research nurses and midwives. Pre-pregnancy weight and height and other prenatal data were ascertained using standardized questionnaire during the OGTT test between 24 and 32 weeks of gestation. A total of 356 women (37.5%) were missing pre-pregnancy weight. We imputed missing pre-pregnancy weight by a multiple imputation procedure (Yuan, 2000) using information from maternal age, education status, height, pre-pregnancy weight, and maternal weight at OGTT, gestational age at OGTT, hypertension and diabetes status. In brief, the missing data were imputed five times followed by analysis of each imputed data using multivariate linear regression. Finally, the results were pooled to produce inferential results. There were no significant differences in the mean and standard deviation of pre-pregnancy weight with or without imputation (65.2 ± 15.2 vs. 65.8 ± 15.7 kg). Maternal ppBMI was calculated as pre-pregnancy weight in kilograms divided by the squared of maternal height in meters. Women were grouped into normal weight (ppBMI < 25 kg/m^2^) and overweight or obese (ppBMI ≥ 25 kg/m^2^) based on their pre-pregnancy BMI.

### Genotyping and Imputation

DNA were genotyped at genome-wide level using the Illumina Human1M-Duo BeadChip as part of the Gene Environment Association Studies initiative; quality control of the genotypes was conducted as reported previously (Urbanek et al., 2013). Genotypes were imputed with the Michigan Imputation Server (Das et al., 2016) implementing Eagle2 (Loh et al., 2016) for haplotype phasing, followed by Minimac2 (Fuchsberger et al., 2015) for imputing non-typed SNPs with 1000 Genomes Phase 3 data (Genomes Project et al., 2015).

### Genetic Risk Score Computation

Genotypes for 97 SNPs associated with adult BMI in a previous large-scale GWAS (Locke et al., 2015) were extracted from the HAPO study to construct a weighted maternal obesity genetic risk score, mGRS and their babies obesity genetic risk score, bGRS. The list of the 97 BMI-associated SNPs along with their effect sizes, and descriptive statistics are shown in Supplementary Table S1 (Locke et al., 2015). The weighted genetic risk score (GRS) was constructed by multiplying the dosage of the BMI-increasing allele for each SNP (range:0–2) by its published effect estimate (Locke et al., 2015), followed by summing the resulting values and multiplying them by the ratio of the sample size and the sum of the effect sizes (Lin et al., 2009).

### Statistical Analyses

The GRSs were analyzed as continuous variables (bGRS and mGRS) and as categorical variables using the median GRS value as a cut-off to define high or low bGRS for babies and high or low mGRS for mothers. To test the association of bGRS with birthweight, multivariate linear regression was used adjusting for maternal age, education, fasting plasma glucose, hypertension status, baby’s gender, gestational age at delivery, the proportion of baby’s African ancestry to account for genetic population structure, without (Model 1a) and with mGRS (Model 2a). We repeated the analyses by adding ppBMI in the list of co-variates to evaluate whether the effect of bGRS on birthweight was independent of maternal pre-pregnancy BMI (Model 3a). When testing for association of mGRS with birthweight, the same models were used substituting mother’s proportion of African ancestry proportion for baby’s proportion of African ancestry and bGRS for mGRS (Models 1b, 2b, 3b).

Additionally, we tested for an interaction between bGRS × mGRS to evaluate whether the effects of bGRS on birthweight varied by mGRS (Model 2a + bGRS × mGRS), and vice versa (Model 2b + bGRS × mGRS). In addition, we evaluated whether the effects of mGRS on birthweight varied by mother’s pre-pregnancy BMI by adding an interaction term to the multivariate linear regression models (i.e., Model 3b + mGRS × pre-pregnancy BMI. The analysis was repeated for categorical (high/low) GRS separately for the babies and mothers. A two-sided *P*-value less than 0.05 was considered to be statistically significant. All analyses were performed using PLINK 1.9 (Chang et al., 2015), and SAS (version 9.4, Cary, NC, United States).

## Results

The characteristics of the mothers and babies included in this study are summarized in Table 1. The mean (±standard deviation) birthweight was 3,220.90 (±452.14) grams, and the mean gestational age at birth was 39.79 (±1.24) weeks. Over 40% of the mothers were overweight or obese (ppBMI ≥ 25 kg/m^2^) based on their pre-pregnancy BMI.

**Table 1:** Characteristics of study participants (*n* = 950 mother-baby pairs).

	Mean ± SD or n (%)
Mother’s age, years	25.51 ± 5.65
Gestational age at birth, weeks	39.79 ± 1.24
Proportion of African ancestry in mothers	0.89 ± 0.11
Proportion of African ancestry in babies	0.86 ± 0.12
Birth weight, g	3,220.90 ± 452.14
Baby sex (female), n (%)	461 (48.53)
**Mother’s education, n (%)**	
Below high school	113 (12.00)
High school	334 (35.16)
No Graduate Degree	467 (49.20)
Graduate or higher	35 (3.64)
Mother’s pre-pregnancy BMI, kg/m^2^	24.52 ± 5.66
**Mother’s pre-pregnancy BMI, kg/m^2^, n (%)**	
<25 kg/m^2^	566 (59.58)
≥25 kg/m^2^	384 (40.42)
Mother’s fasting plasma glucose, mg/dl	80.59 ± 7.78
**Hypertension in mothers, n (%)**	
None	872 (91.79)
Yes	78 (8.21)
bGRS	52.89 ± 2.75
bGRS (below median)	50.73 ± 1.72
bGRS (median or above)	55.07 ± 1.64
mGRS	52.73 ± 2.72
mGRS (below median)	50.57 ± 1.73
mGRS (median or above)	54.94 ± 1.52

*bGRS, Obesity genetic risk score in babies; mGRS, Obesity genetic risk score in mothers.*

The associations of bGRS and mGRS with birthweight are presented in Tables 2, 3. A unit increase in bGRS was significantly associated with 13.05 g lower birthweight (95% CI = −24.70 to −1.40, *P* = 0.03). In categorical analysis of bGRS, high bGRS was significantly associated with 72.49 g lower birthweight (95% CI = −130.04 to −14.95, *P* = 0.01) compared to low bGRS (Table 2). Conversely, mGRS appeared to have positive relationship with birthweight, although the association was not statistically significant (Table 3). Further adjustment for maternal ppBMI (model 3) did not substantially change the above association results (Tables 2, 3). Full Model including other covariates are shown in Supplementary Tables S2, S3.

**Table 2:** Association of fetal obesity genetic risk with birthweight.

	Model 1a^a^		Model 2a^b^		Model 3a^c^	
	β (95% CI)	*P*	β (95% CI)	*P*	β (95% CI)	*P*
**bGRS**	−8.84 (−18.82, 1.14)	0.08	−13.05 (−24.70, −1.40)	0.03	−12.66 (−24.30, −1.02)	0.03
**High bGRS vs. Low bGRS (Ref)**	−68.00 (−122.58, −13.42)	0.01	−72.49 (−130.04, −14.95)	0.01	−69.90 (−127.41, −12.38)	0.01

*^a^Model 1a included bGRS, maternal age, education, fasting plasma glucose (mg/dl), hypertension status, baby’s gender, gestational age at delivery, and baby’s proportion of African ancestry; ^b^Model 2a included Model 1a and mGRS; ^c^Model 3a included Model 2a and maternal pre-pregnancy BMI. bGRS, Obesity genetic risk score in babies; mGRS, Obesity genetic risk score in mothers.*

**Table 3:** Association of maternal obesity genetic risk with birthweight.

	Model 1b^a^		Model 2b^b^		Model 3b^c^	
	β (95% CI)	*P*	β (95% CI)	*P*	β (95% CI)	*P*
**mGRS**	1.15 (−8.91, 11.21)	0.82	7.80 (−3.93, 19.53)	0.19	7.11 (−4.62, 18.84)	0.23
**High mGRS vs. Low mGRS (Ref)**	−9.09 (−63.71, 45.53)	0.75	13.83 (−43.58, 71.23)	0.64	12.17 (−45.16, 69.50)	0.67

*^a^Model 1b included mGRS, maternal age, education, fasting plasma glucose (mg/dl), hypertension status, baby’s gender, gestational age at delivery, and mother’s proportion of African ancestry; ^b^Model 2b included Model 1b + bGRS; ^c^Model 3b included Model 2b + maternal pre-pregnancy BMI. bGRS, Obesity genetic risk score in babies; mGRS, Obesity genetic risk score in mothers.*

We observed borderline significant interaction between bGRS and mGRS in relation to birthweight (*P-for-interaction* = 0.06). When stratifying further by categorical mGRS, the birthweight-lowering effect of bGRS strengthened and was statistically significant among mothers with high genetic risk for obesity (β = −20.06, 95%CI = −35.59 to −4.54, *P* = 0.01). Compared to low bGRS, high bGRS was associated with 114.11 g lower birthweight (95% CI = −194.96 to −33.26, *P* = 0.005) among mothers with high genetic risk for obesity. No such effect was seen among mothers with low genetic risk for obesity (Table 4). When stratified by categorical bGRS, the birthweight-increasing effect of mGRS appeared to be stronger among babies with low genetic risk for obesity, however, the associations were not statistically significant (Table 5).

**Table 4:** Association of fetal obesity genetic risk with birthweight stratified by maternal obesity genetic risk status^a^.

	Low mGRS (*n* = 480)		High mGRS (*n* = 470)		*P-for-interaction*
	β (95% CI)	*P*	β (95% CI)	*P*	
**bGRS**	0.57 (−14.70, 15.85)	0.94	−20.06 (−35.59, −4.54)	0.01	0.06
**High bGRS vs. Low bGRS (Ref)**	−24.11 (−105.77, 57.54)	0.56	−114.11 (−194.96, −33.26)	0.005	0.12

*^a^adjusted for maternal age, education, fasting plasma glucose (mg/dl), hypertension status, baby’s gender, gestational age at delivery, and baby’s proportion of African ancestry. mGRS, Obesity genetic risk score in mothers; bGRS, Obesity genetic risk score in babies.*

**Table 5:** Association of maternal obesity genetic risk with birthweight stratified by fetal obesity genetic risk status^a^.

	Low bGRS (*n* = 476)		High bGRS (*n* = 474)		*P-for-interaction*
	β (95% CI)	*P*	β (95% CI)	*P*	
**mGRS**	13.91 (−0.90, 30.03)	0.06	−1.11 (−17.10, 14.89)	0.89	0.14
**High mGRS vs. Low mGRS (Ref)**	64.92 (−16.59, 146.42)	0.11	−26.65 (−107.42, 54.12)	0.54	0.10

*^a^adjusted for maternal age, education, fasting plasma glucose (mg/dl), hypertension status, baby’s gender, gestational age at delivery, and mother’s proportion of African ancestry. mGRS, Obesity genetic risk score in mothers; bGRS, Obesity genetic risk score in babies.*

As expected, mother’s ppBMI was positively and significantly associated with birthweight for continuous ppBMI (β = 5.63, 95% CI = 0.29 to 10.95, *P* = 0.03). Similarly, babies of mothers with ppBMI ≥ 25 kg/m^2^ were 71.26 g heavier (95% CI = −2.60 to 145.11, *P* = 0.05) than babies of mothers with ppBMI < 25 kg/m^2^. We observed significant interactions of categorical mGRS (high vs. low) and ppBMI (≥25 kg/m^2^ vs. <25 kg/m^2^ in relation to birthweight (*P-for-interaction* = 0.03). The birthweight-increasing effect of ppBMI was found to be stronger and significant among mothers with low genetic risk for obesity (β = 8.13, 95% CI = 0.57 to 15.68, *P* = 0.03). Similarly, compared to babies of mothers with ppBMI < 25 kg/m^2^, babies of mothers with ppBMI ≥ 25 kg/m^2^ were 126.86 g heavier (95% CI = 25.81 to 227.91, *P* = 0.01) among mothers with low genetic risk for obesity. The birthweight-increasing effects of ppBMI were weaker and not significant among mothers with high genetic risk for obesity (Table 6).

**Table 6:** Association of maternal pre-pregnancy body mass index with birthweight stratified by maternal GRS.

	Overall sample^a^ (*n* = 950)		Low mGRS^b^ (*n* = 480)		High mGRS^b^ (*n* = 470)		*P-for-interaction*
	β (95% CI)	*P*	β (95% CI)	*P*	β (95% CI)	*P*	
**Pre-pregnancy BMI, kg/m^2^**	5.62 (0.29, 10.95)	0.03	8.13 (0.57, 15.68)	0.03	3.16 (−4.66, 10.98)	0.42	0.29
**Pre-pregnancy BMI status**							
**≥25 kg/m^2^ vs. <25 kg/m^2^ (Ref)**	71.26 (−2.60, 145.11)	0.05	126.86 (25.81, 227.91)	0.01	18.85 (−73.35, 111.05)	0.68	0.03

*^a^adjusted for maternal age, education, fasting plasma glucose (mg/dl), hypertension status, baby’s gender, gestational age at delivery, mGRS, bGRS, and mother’s proportion of African ancestry. ^b^adjusted for maternal age, education, fasting plasma glucose (mg/dl), hypertension status, baby’s gender, gestational age at delivery, bGRS, and mother’s proportion of African ancestry. mGRS, Obesity genetic risk score in mothers; bGRS, Obesity genetic risk score in babies.*

## Discussion

Using 950 mother-offspring pairs of African ancestry, we investigated the influence of genetic susceptibility to adulthood obesity on offspring birthweight. The study revealed three key findings. First, fetal genetic risk to adulthood obesity exerted and significant birthweight-lowering effect. Second, maternal genetic risk to obesity exerted weak birthweight-increasing effect that did not reach statistical significance, but it modified the effect of fetal obesity genetic susceptibility on birthweight. Specifically, fetal genetic susceptibility to obesity showed stronger and significant inverse association with birthweight when the mother’s genetic susceptibility to obesity is high. Third, maternal genetic risk modified the effect of maternal pre-pregnancy BMI. Specifically, the birthweight-increasing effect of pre-pregnancy BMI doubled when the mother’s genetic susceptibility to adulthood obesity was low.

Birthweight has a special implication for early origin of adulthood adiposity as offspring born with a low birthweight tend to have a more visceral distribution of obesity and significantly reduced muscle mass (Budge et al., 2009; Estampador and Franks, 2014; Ribeiro et al., 2015). The biological processes that link lower birthweight with rapid post-natal catch-up growth, early-onset adiposity rebound (i.e., rise in BMI after infancy) in childhood, and increased risk of obesity in adulthood are not clearly understood. A faster tempo of childhood growth has been found to be associated with higher risk of adulthood obesity (Parsons et al., 2001; Stovitz et al., 2011). Children with higher BMI GRS experience adiposity rebound at an earlier age and with a higher BMI (Warrington et al., 2013). Higher BMI GRS was also positively associated with higher (Elks et al., 2010) and more rapid weight gain (Belsky et al., 2012) during early post-natal life and with body weight across childhood and adulthood, particularly after age 2–3 years (Elks et al., 2010; Belsky et al., 2012). The present study’s finding that higher fetal BMI GRS has birthweight-lowering effect suggests that the *in utero* effects of obesity genetic risk loci on fetal growth are different from their post-natal effects on childhood weight.

Published studies investigating association of adult obesity GRS with birthweight were carried out using only fetal genotypes. Most of those studies reported null associations between fetal obesity GRS and birthweight (Andersson et al., 2010;Elks et al., 2010, 2014; Kilpelaeinen et al., 2011; Belsky et al., 2012; Warrington et al., 2013). The differences among these studies could mainly be due to differences in the number of SNPs included in the calculation of the GRS, which ranged from 8 SNPs (Elks et al., 2010) to 32 SNPs (Belsky et al., 2012; Warrington et al., 2013) across studies. In addition, the studies did not involve samples from non-European ancestry populations. In a previous multi-ethnic population study of samples from the HAPO study, SNPs from 40 genomic loci known to be associated with adult BMI or waist-to-hip-ratio were tested for association with birthweight (Chawla et al., 2014). Twelve out of the 40 genetic loci were significantly associated with birthweight, and a GRS composed of these 12 SNPs was positively associated with birthweight (Chawla et al., 2014). However, the 12 SNPs included in the estimation of GRS were not associated with adult BMI or waist-to-hip ratio (Chawla et al., 2014), implying that the GRS used in the association analysis does not represent genetic risk to adulthood obesity.

The GRS for adulthood obesity estimated in our study was more comprehensive than the previous studies because it was based on 2–5 times more BMI genetic risk loci (total 97 SNPs) compared to previous studies and also involved both fetal and maternal genotypes. Consistent with our finding, in a study of more than 28,219 Europeans, an obesity GRS formed from 12 SNPs has been associated with 3 g decreased birthweight, although it was short of statistical significance (Kilpelaeinen et al., 2011). The study also found that BMI-increasing alleles in the *MTCH2* gene have been inversely associated with birthweight (Kilpelaeinen et al., 2011). Two other studies using GRS of 11 and 83 BMI-associated variants found suggestive (Elks et al., 2012b), and statistically significant positive association (Li et al., 2017) with birthweight and birthweight Z-score, respectively. The number of SNPs used to generate the GRS in both of the studies was smaller than our study’s. More importantly, both studies did not adjust for maternal GRS while testing for the effect of fetal GRS on birthweight. Given the directionally opposite effects of maternal and fetal obesity GRS as observed in the present study, failure to account for maternal GRS is likely to confound the effect of fetal GRS on birthweight.

No genetic studies have investigated the association between genetic risk of obesity and birthweight longitudinally throughout the life course. However, some BMI genetic variants (such as *PCSK1* and *OLFM4*) have been found to bear stronger effect on weight during infancy or childhood than adulthood (Elks et al., 2012a; Pigeyre et al., 2016; Rukh et al., 2016). Shared genetic effect for change in BMI has also been found to be age and sex dependent (North et al., 2010; Elks et al., 2012a; Winkler et al., 2015). Our findings further suggest that children born smaller consequent to higher BMI GRS are very likely to experience stronger adiposity rebound effects because of compensatory catch-up growth following smaller birth size as well as increased adiposity rebound effects of the birthweight-lowering BMI loci.

To our knowledge, our study is the first to show that maternal genetic susceptibility to obesity modifies the association between fetal genetic susceptibility to obesity and birthweight and the association between maternal pre-pregnancy BMI and birthweight (Li et al., 2017). Understanding the intra-uterine pathways that mediate the effects of maternal BMI GRS on birthweight opens a new avenue to develop interventions to avert unwanted effects of pregnancies at high genetic risk of aberrant fetal growth. Obviously, optimal nutritional availability, gestational diabetes or gestational weight gain (Lawlor et al., 2011) account for long-term programming of adiposity in intrauterine environment. The effect of nutrient availability in the intrauterine environment could alter birthweight through different pathways (Catalano and Ehrenberg, 2006; Castillo et al., 2015). Our finding that the effect of GRS on birthweight was independent of ppBMI indicates that future investigations are needed to understand the intra-uterine pathways that mediate the effect of maternal BMI GRS on birthweight.

This study has several strengths. The GRS of adulthood obesity was derived from a comprehensive list of 97 SNPs established to be associated with BMI (Locke et al., 2015). Second, the study included fetal and maternal genetic loci, incorporating genetic ancestry information in a well-characterized study population. Third, the study involved African Ancestry individuals who were under-represented in GWAS of complex traits, despite high prevalence (>30%) of obesity, disproportionately affecting this population (Adams et al., 2006). However, several limitations of this study deserve mention. We calculated GRSs using SNPs associated with BMI at genome wide significant level in European ancestry populations, and not in African-ancestry populations. Despite the limitation that allele frequencies and patterns of linkage disequilibrium vary across ancestral populations, emerging evidence shows that cumulative information across the human genome can be used to characterize individual level risk for obesity with small variation among Whites and Blacks supporting the use of GRS in transethnic studies (Domingue et al., 2014). Absence of data on gestational weight at different trimesters of pregnancy, an important determinant of birthweight (Pugh et al., 2017), limited our ability to evaluate whether the effects of mGRS and bGRS are modified by weight gain during pregnancy.

## Conclusion

The present study found that fetal genetic risk of adulthood obesity had strong birthweight-lowering effect. Conversely, maternal genetic risk of adulthood obesity had modified the associations between pre-pregnancy BMI and birthweight. Further elucidation of these associations and incorporation of genetic information may provide important scientific, clinical and public health insights to optimize neonatal health and to curb the trans-generational cycle of obesity. In general, individual SNP effects are small; however, summarizing the effects in GRS has been demonstrated to predict individual’s overall risk to disease and showed promises in the field of cardiac disease to revolutionize medicine on multiple levels (Torkamani et al., 2018; Zheutlin and Ross, 2018). Obesity, being a multifactorial hereditary disorder, detailed characterization of interactions between GRS and modifiable environmental factors will facilitate the development of targeted preventative lifestyle, nutritional and clinical interventions.

## Data Availability Statement

The data used in our analysis is available through dbGaP (https://www.ncbi.nlm.nih.gov/gap).

## Author Contributions

FT-A conceived and designed this study. DS and CZ performed the statistical analyses. DS and FT-A wrote the draft manuscript. All authors contributed to interpretation of the results, provided critical intellectual content, and approved the final manuscript.

## Conflict of Interest Statement

The authors declare that the research was conducted in the absence of any commercial or financial relationships that could be construed as a potential conflict of interest.
