# Supplementary material for: Influence of Fetal and Maternal Genetic Susceptibility to Obesity on Birthweight in African Ancestry Populations
Source: Front Genet. 2018 Nov 2;9:511. doi: 10.3389/fgene.2018.00511 (PMC6224338; doi:10.3389/fgene.2018.00511)

**Influence of fetal and maternal genetic susceptibility to obesity on birthweight in African ancestry populations**

Deepika Shrestha^1^, Mohammad Rahman^1^, Tsegaselassie Workalemahu^1^, Chunming Zhu^2^, Fasil Tekola-Ayele^1*^

^1^ Epidemiology Branch, Division of Intramural Population Health Research, *Eunice Kennedy Shriver* National Institute of Child Health and Human Development, National Institutes of Health, Bethesda, MD, USA. ^2^ Division of Intramural Population Health Research, *Eunice Kennedy Shriver* National Institute of Child Health and Human Development, National Institutes of Health, Bethesda, MD, USA

***Correspondence:**

Fasil Tekola-Ayele, Ph.D.

Epidemiology Branch, Division of Intramural Population Health Research, *Eunice Kennedy Shriver* National Institute of Child Health and Human Development, National Institutes of Health, 6710B Rockledge Dr, 6710B-3204, Bethesda, Maryland 20892-7004

Email: [ayeleft@mail.nih.gov](mailto:ayeleft@mail.nih.gov)

Tel: 301-827-6518

**Table S1. List of the 97 BMI-associated SNPs used to create a genetic risk score for obesity**

| **SNP** | **Chr_hg19position** | **Chr. Id** | **Gene** | **Risk Allele**  **(increasing BMI)** | **Alternate Allele** | **Effect size** | ***P* value** | ***Reference Allele*** | ***Allele Frequency in Africans*** | ***Allele Frequency***  ***in Europeans*** |
| --- | --- | --- | --- | --- | --- | --- | --- | --- | --- | --- |
| rs1000940 | 17:5379957 | 17 | RABEP1 | G | A | 0.3199 | 1.28E-08 | A | 0.23 | 0.3489 |
| rs10132280 | 14:24998019 | 14 | STXBP6 | C | A | 0.6816 | 1.14E-11 | C | 0.5507 | 0.3221 |
| rs1016287 | 2:59078490 | 2 | LINC011 | T | C | 0.2867 | 2.25E-11 | T | 0.8033 | 0.7197 |
| rs10182181 | 2:25003800 | 2 | ADCY3 | G | A | 0.4617 | 8.78E-24 | A | 0.9123 | 0.4662 |
| rs10733682 | 9:128500735 | 9 | LMX1B | A | G | 0.4782 | 1.83E-08 | A | 0.7519 | 0.494 |
| rs10938397 | 4:44877284 | 4 | GNPDA2 | G | A | 0.4341 | 3.21E-38 | A | 0.2042 | 0.4205 |
| rs10968576 | 9:28404339 | 9 | LINGO2 | G | A | 0.32 | 6.61E-14 | A | 0.1422 | 0.3022 |
| rs11030104 | 11:27641093 | 11 | BDNF | A | G | 0.7923 | 5.56E-28 | A | 0.0189 | 0.2207 |
| rs11057405 | 12:121347850 | 12 | CLIP1 | G | A | 0.9013 | 2.02E-08 | G | 0.0015 | 0.0895 |
| rs11126666 | 2:26782315 | 2 | KCNK3 | A | G | 0.2833 | 1.33E-09 | G | 0.174 | 0.2714 |
| rs11165643 | 1:96696685 | 1 | PTBP2 | T | C | 0.583 | 2.07E-12 | C | 0.1225 | 0.5825 |
| rs11191560 | 10:104859028 | 10 | NT5C2 | C | T | 0.0891 | 8.45E-09 | T | 0.0219 | 0.0875 |
| rs11583200 | 1:50332407 | 1 | ELAVL4 | C | T | 0.3958 | 1.48E-08 | C | 0.2307 | 0.6272 |
| rs1167827 | 7:75533848 | 7 | HIP1 | G | A | 0.5526 | 6.33E-10 | G | 0.0385 | 0.4602 |
| rs11688816 | 2:62906552 | 2 | EHBP1 | G | A | 0.5246 | 1.89E-08 | G | 0.3245 | 0.5318 |
| rs11727676 | 4:145878514 | 4 | HHIP | T | C | 0.9101 | 2.55E-08 | T | 0.0023 | 0.0765 |
| rs11847697 | 14:29584863 | 14 | PRKD1 | T | C | 0.042 | 3.99E-09 | C | 0.4092 | 0.0517 |
| rs12016871 | 13:26915782 | 13 | MTIF3 | T | C | 0.2029 | 2.29E-10 | C | 0.95 | 0.78 |
| rs12286929 | 11:114527614 | 11 | CADM1 | G | A | 0.5229 | 1.31E-12 | A | 0.5802 | 0.4841 |
| rs12401738 | 1:78219349 | 1 | FUBP1 | A | G | 0.352 | 1.15E-10 | G | 0.0605 | 0.3002 |
| rs12429545 | 13:53000207 | 13 | OLFM4 | A | G | 0.1334 | 1.09E-12 | G | 0.0257 | 0.1113 |
| rs12446632 | 16:19842890 | 16 | GPRC5B | G | A | 0.865 | 1.48E-18 | G | 0.0825 | 0.1302 |
| rs12566985 | 1:74774781 | 1 | FPGT | G | A | 0.4459 | 3.28E-15 | G | 0.1702 | 0.5467 |
| rs12885454 | 14:28806589 | 14 | PRKD1 | C | A | 0.6416 | 1.94E-10 | C | 0.0893 | 0.338 |
| rs12940622 | 17:76230166 | 17 | RPTOR | G | A | 0.5748 | 2.49E-09 | G | 0.5681 | 0.4324 |
| rs13021737 | 2:622348 | 2 | TMEM18 | G | A | 0.828 | 1.11E-50 | A | 0.9047 | 0.826 |
| rs13078960 | 3:85890280 | 3 | CADM2 | G | T | 0.1955 | 1.74E-14 | T | 0.0424 | 0.2078 |
| rs13107325 | 4:103407732 | 4 | SLC39A8 | T | C | 0.0722 | 1.83E-12 | C | 0.0023 | 0.0795 |
| rs13191362 | 6:162953340 | 6 | PARK2 | A | G | 0.8791 | 7.34E-09 | A | 0.0348 | 0.1282 |
| rs13201877 | 6:137717234 | 6 | IFNGR1 | G | A | 0.1421 | 2.35E-07 | A | 0.0182 | 0.1292 |
| rs1441264 | 13:79006784 | 13 | MIR548A | A | G | 0.6092 | 6.04E-08 | G | 0.7352 | 0.6262 |
| rs1460676 | 2:163711179 | 2 | FIGN | C | T | 0.173 | 8.98E-07 | T | 0.2595 | 0.169 |
| rs1516725 | 3:186106215 | 3 | ETV5 | C | T | 0.8719 | 1.89E-22 | T | 0.8321 | 0.8618 |
| rs1528435 | 2:180686235 | 2 | UBE2E3 | T | C | 0.6312 | 1.20E-08 | C | 0.6203 | 0.5845 |
| rs1558902 | 16:53769662 | 16 | FTO | A | T | 0.415 | 7.51E-153 | T | 0.056 | 0.4324 |
| rs16851483 | 3:142758126 | 3 | RASA2 | T | G | 0.0655 | 3.55E-10 | G | 0.003 | 0.0646 |
| rs16907751 | 8:81538012 | 8 | ZBTB10 | C | T | 0.9156 | 1.26E-07 | C | 0.0673 | 0.1402 |
| rs16951275 | 15:65864222 | 15 | MAP2K5 | T | C | 0.7841 | 1.91E-17 | T | 0.4138 | 0.2366 |
| rs17001654 | 4:77348592 | 4 | SCARB2 | G | C | 0.1532 | 7.76E-09 | C | 0.1657 | 0.173 |
| rs17024393 | 1:109956211 | 1 | GNAT2 | C | T | 0.0397 | 7.03E-14 | T | 0.084 | 0.0328 |
| rs17094222 | 10:102385430 | 10 | HIF1AN | C | T | 0.2106 | 5.94E-11 | T | 0.0265 | 0.1968 |
| rs17203016 | 2:207963763 | 2 | CREB1 | G | A | 0.197 | 8.15E-08 | A | 0.0121 | 0.1869 |
| rs17405819 | 8:76969139 | 8 | HNF4G | T | C | 0.7 | 2.07E-11 | T | 0.0287 | 0.3042 |
| rs17724992 | 19:18315825 | 19 | PGPEP1 | A | G | 0.7456 | 3.42E-08 | A | 0.09 | 0.2445 |
| rs1808579 | 18:23524924 | 18 | C18orf8 | C | T | 0.5339 | 4.17E-08 | C | 0.4569 | 0.4473 |
| rs1928295 | 9:117616205 | 9 | TLR4 | T | C | 0.5479 | 7.91E-10 | T | 0.4501 | 0.4423 |
| rs2033529 | 6:40380914 | 6 | TDRG1 | G | A | 0.293 | 1.39E-08 | A | 0.09 | 0.2763 |
| rs2033732 | 8:84167474 | 8 | RALYL | C | T | 0.7469 | 4.89E-08 | T | 0.9123 | 0.7664 |
| rs205262 | 6:34595387 | 6 | C6orf10 | G | A | 0.273 | 1.75E-10 | A | 0.7292 | 0.2793 |
| rs2075650 | 19:44892362 | 19 | TOMM40 | A | G | 0.8484 | 1.25E-08 | A | 0.1309 | 0.1312 |
| rs2080454 | 16:49028679 | 16 | CBLN1 | C | A | 0.4059 | 6.55E-08 | C | 0.2814 | 0.6203 |
| rs2112347 | 5:75719417 | 5 | POC5 | T | G | 0.6289 | 6.19E-17 | T | 0.5234 | 0.3678 |
| rs2121279 | 2:142285716 | 2 | LRP1B | T | C | 0.152 | 2.31E-08 | C | 0.0121 | 0.1312 |
| rs2176040 | 2:226228086 | 2 | LOC6467 | A | G | 0.3654 | 6.06E-06 | A | 0.6846 | 0.6262 |
| rs2176598 | 11:43842728 | 11 | HSD17B1 | T | C | 0.251 | 2.97E-08 | T | 0.621 | 0.7376 |
| rs2207139 | 6:50877777 | 6 | TFAP2B | G | A | 0.1773 | 4.13E-29 | A | 0.0787 | 0.164 |
| rs2245368 | 7:76978826 | 7 | DTX2P1 | C | T | 0.1798 | 3.19E-08 | C | 0.8533 | 0.7913 |
| rs2287019 | 19:45698914 | 19 | QPCTL | C | T | 0.8038 | 4.59E-18 | C | 0.121 | 0.1968 |
| rs2365389 | 3:61250788 | 3 | FHIT | C | T | 0.5816 | 1.63E-10 | C | 0.8654 | 0.3897 |
| rs2650492 | 16:28322090 | 16 | SBK1 | A | G | 0.3033 | 1.92E-09 | G | 0.0106 | 0.2386 |
| rs2820292 | 1:201815159 | 1 | NAV1 | C | A | 0.555 | 1.83E-10 | A | 0.3124 | 0.5398 |
| rs2836754 | 21:38919816 | 21 | ETS2 | C | T | 0.6116 | 4.16E-07 | T | 0.3366 | 0.6153 |
| rs29941 | 19:33818627 | 19 | KCTD15 | G | A | 0.6685 | 2.41E-08 | A | 0.8525 | 0.667 |
| rs3101336 | 1:72285502 | 1 | NEGR1 | C | T | 0.6125 | 2.66E-26 | T | 0.5303 | 0.6412 |
| rs3736485 | 15:51456413 | 15 | DMXL2 | A | G | 0.454 | 7.41E-09 | A | 0.3722 | 0.5348 |
| rs3810291 | 19:47065746 | 19 | ZC3H4 | A | G | 0.6664 | 4.81E-15 | G | 0.0893 | 0.663 |
| rs3817334 | 11:47629441 | 11 | MTCH2 | T | C | 0.4067 | 5.15E-17 | C | 0.2126 | 0.4215 |
| rs3849570 | 3:81742961 | 3 | GBE1 | A | C | 0.3593 | 2.60E-08 | C | 0.4977 | 0.328 |
| rs3888190 | 16:28878165 | 16 | ATP2A1 | A | C | 0.4025 | 3.14E-23 | C | 0.2284 | 0.33 |
| rs4256980 | 11:8652392 | 11 | TRIM66 | G | C | 0.6462 | 2.90E-11 | C | 0.497 | 0.6272 |
| rs4740619 | 9:15634328 | 9 | C9orf93 | T | C | 0.5416 | 4.56E-09 | T | 0.4448 | 0.4811 |
| rs4787491 | 16:30004016 | 16 | INO80E | G | A | 0.5085 | 2.24E-06 | A | 0.5204 | 0.5338 |
| rs492400 | 2:218485029 | 2 | USP37 | C | T | 0.4234 | 4.17E-07 | C | 0.4259 | 0.5924 |
| rs543874 | 1:177920345 | 1 | SEC16B | G | A | 0.1927 | 2.62E-35 | A | 0.2761 | 0.1869 |
| rs6091540 | 20:52471323 | 20 | ZFP64 | C | T | 0.7227 | 8.02E-08 | C | 0.2171 | 0.2714 |
| rs6465468 | 7:95540202 | 7 | ASB4 | T | G | 0.304 | 2.32E-06 | G | 0.1218 | 0.3032 |
| rs6477694 | 9:109170062 | 9 | EPB41L4 | C | T | 0.3653 | 2.67E-08 | C | 0.5968 | 0.672 |
| rs6567160 | 18:60161902 | 18 | MC4R | C | T | 0.2357 | 3.93E-53 | T | 0.2201 | 0.2396 |
| rs657452 | 1:49124175 | 1 | AGBL4 | A | G | 0.394 | 5.48E-13 | A | 0.4206 | 0.6252 |
| rs6804842 | 3:25064946 | 3 | RARB | G | A | 0.5749 | 2.48E-09 | A | 0.3563 | 0.5626 |
| rs7138803 | 12:49853685 | 12 | BCDIN3D | A | G | 0.3843 | 8.15E-24 | G | 0.1331 | 0.338 |
| rs7141420 | 14:79433111 | 14 | NRXN3 | T | C | 0.5267 | 1.23E-14 | C | 0.6172 | 0.5298 |
| rs7164727 | 15:72801650 | 15 | LOC1002 | T | C | 0.6864 | 6.83E-08 | C | 0.27 | 0.6958 |
| rs7239883 | 18:42567706 | 18 | LOC2842 | G | A | 0.3934 | 1.63E-07 | G | 0.5492 | 0.5835 |
| rs7243357 | 18:59216087 | 18 | GRP | T | G | 0.8117 | 3.86E-08 | T | 0.1256 | 0.1879 |
| rs758747 | 16:3577357 | 16 | NLRC3 | T | C | 0.2651 | 7.47E-10 | C | 0.6324 | 0.2803 |
| rs7599312 | 2:212548507 | 2 | ERBB4 | G | A | 0.7238 | 1.17E-10 | G | 0.4198 | 0.2654 |
| rs7715256 | 5:154158333 | 5 | GALNT10 | G | T | 0.4211 | 1.70E-07 | G | 0.6437 | 0.5586 |
| rs7899106 | 10:85651147 | 10 | GRID1 | G | A | 0.0517 | 2.96E-08 | A | 0.1452 | 0.0408 |
| rs7903146 | 10:112998590 | 10 | TCF7L2 | C | T | 0.7129 | 1.11E-11 | C | 0.2602 | 0.3171 |
| rs9374842 | 6:119864519 | 6 | LOC2857 | T | C | 0.748 | 9.67E-08 | C | 0.7814 | 0.7376 |
| rs9400239 | 6:108656460 | 6 | FOXO3 | C | T | 0.6879 | 1.61E-08 | T | 0.1641 | 0.6322 |
| rs9540493 | 13:65631572 | 13 | MIR548X | A | G | 0.4559 | 1.42E-07 | A | 0.3638 | 0.5348 |
| rs9641123 | 7:93568420 | 7 | CALCR | C | G | 0.4285 | 5.00E-07 | G | 0.0265 | 0.4046 |
| rs977747 | 1:47219005 | 1 | TAL1 | T | G | 0.3913 | 8.65E-08 | T | 0.3033 | 0.6054 |
| rs9914578 | 17:2101842 | 17 | SMG6 | G | C | 0.211 | 8.99E-08 | C | 0.6014 | 0.1928 |
| rs9925964 | 16:31118574 | 16 | KAT8 | A | G | 0.6198 | 8.11E-10 | A | 0.0885 | 0.3817 |

**Table S2. Association of fetal obesity genetic risk with birthweight**

| **Fetal GRS** |  | **Model 1a ^a^** | | | |  | **Model 2a ^b^** | | | |  | **Model 3a ^c^** | | | |
| --- | --- | --- | --- | --- | --- | --- | --- | --- | --- | --- | --- | --- | --- | --- | --- |
|  |  | ***β*** | **(95% CI)** | | ***P*** |  | ***β*** | **(95% CI)** | | ***P*** |  | ***β*** | **(95% CI)** | | ***P*** |
| **Continuous GRS** | | | | | | | | | | | | | | | |
| bGRS |  | -8.84 | -18.82 | 1.14 | 0.08 |  | -13.05 | -24.70 | -1.40 | 0.03 |  | -12.66 | -24.30 | -1.02 | 0.03 |
| mGRS |  |  |  |  |  |  | 8.21 | -3.53 | 19.96 | 0.17 |  | 7.52 | -4.23 | 19.26 | 0.21 |
| ppBMI |  |  |  |  |  |  |  |  |  |  |  | 5.66 | 0.32 | 11.00 | 0.04 |
| Education | Graduate or higher | 155.51 | -8.11 | 319.13 | 0.06 |  | 159.18 | -4.44 | 322.80 | 0.06 |  | 166.06 | 2.60 | 329.53 | 0.05 |
|  | No Graduate Degree | -6.59 | -95.57 | 82.38 | 0.88 |  | -5.83 | -94.75 | 83.08 | 0.90 |  | -4.64 | -93.65 | 84.37 | 0.92 |
|  | High school | -65.28 | -157.39 | 26.83 | 0.16 |  | -64.90 | -156.95 | 27.16 | 0.17 |  | -64.07 | -156.09 | 27.94 | 0.17 |
|  | Below high school | *ref* | *ref* | *ref* | *ref* |  | *ref* | *ref* | *ref* | *ref* |  | *ref* | *ref* | *ref* | *ref* |
| Hypertension | Yes | -45.60 | -146.59 | 55.40 | 0.38 |  | -42.34 | -143.39 | 58.72 | 0.41 |  | -67.49 | -171.19 | 36.20 | 0.20 |
|  | No | *ref* | *ref* | *ref* | *ref* |  | *ref* | *ref* | *ref* | *ref* |  | *ref* | *ref* | *ref* | *ref* |
| Gender | Female | -132.17 | -187.03 | -77.30 | <.0001 |  | -132.12 | -186.96 | -77.28 | <.0001 |  | -132.48 | -187.24 | -77.72 | <.0001 |
|  | Male | *ref* | *ref* | *ref* | *ref* |  | *ref* | *ref* | *ref* | *ref* |  | *ref* | *ref* | *ref* | *ref* |
| Age |  | 6.82 | 1.83 | 11.80 | 0.01 |  | 6.62 | 1.64 | 11.61 | 0.01 |  | 5.84 | 0.81 | 10.87 | 0.02 |
| Fasting glucose |  | 8.40 | 4.76 | 12.04 | <.0001 |  | 8.36 | 4.72 | 11.99 | <.0001 |  | 7.19 | 3.40 | 10.98 | 0.00 |
| Gestation age |  | 80.01 | 58.04 | 101.98 | <.0001 |  | 79.13 | 57.13 | 101.12 | <.0001 |  | 76.87 | 54.80 | 98.94 | <.0001 |
| African ancestry |  | -119.92 | -359.07 | 119.24 | 0.33 |  | -117.87 | -356.93 | 121.19 | 0.33 |  | -120.19 | -358.84 | 118.46 | 0.32 |
| **Categorical GRS** | | | | | | | | | | | | | | | |
| bGRS (high vs low) |  | -68.00 | -122.58 | -13.42 | 0.01 |  | -72.49 | -130.04 | -14.95 | 0.01 |  | -69.90 | -127.41 | -12.38 | 0.02 |
| mGRS (high vs low) |  |  |  |  |  |  | 14.23 | -43.31 | 71.77 | 0.63 |  | 12.57 | -44.89 | 70.03 | 0.67 |
| ppBMI |  |  |  |  |  |  |  |  |  |  |  | 5.60 | 0.26 | 10.94 | 0.04 |
| Education | Graduate or higher | 154.78 | -8.58 | 318.13 | 0.06 |  | 155.24 | -8.18 | 318.67 | 0.06 |  | 162.35 | -0.94 | 325.64 | 0.05 |
|  | No Graduate Degree | -10.56 | -99.27 | 78.15 | 0.82 |  | -10.61 | -99.34 | 78.12 | 0.81 |  | -9.32 | -98.14 | 79.49 | 0.84 |
|  | High school | -68.76 | -160.77 | 23.25 | 0.14 |  | -68.50 | -160.54 | 23.54 | 0.14 |  | -67.53 | -159.53 | 24.47 | 0.15 |
|  | Below high school | *ref* | *ref* | *ref* | *ref* |  | *ref* | *ref* | *ref* | *ref* |  | *ref* | *ref* | *ref* | *ref* |
| Hypertension | Yes | -48.12 | -148.87 | 52.63 | 0.35 |  | -47.05 | -147.93 | 53.83 | 0.36 |  | -71.82 | -175.32 | 31.67 | 0.17 |
|  | No | *ref* | *ref* | *ref* | *ref* |  | *ref* | *ref* | *ref* | *ref* |  | *ref* | *ref* | *ref* | *ref* |
| Gender | Female | -133.47 | -188.23 | -78.71 | <.0001 |  | -133.82 | -188.62 | -79.03 | <.0001 |  | -134.14 | -188.86 | -79.41 | <.0001 |
|  | Male | *ref* | *ref* | *ref* | *ref* |  | *ref* | *ref* | *ref* | *ref* |  | *ref* | *ref* | *ref* | *ref* |
| Age |  | 6.79 | 1.81 | 11.76 | 0.01 |  | 6.73 | 1.74 | 11.71 | 0.01 | . | 5.94 | 0.91 | 10.97 | 0.02 |
| Fasting glucose |  | 8.57 | 4.93 | 12.21 | <.0001 |  | 8.56 | 4.93 | 12.20 | <.0001 |  | 7.40 | 3.61 | 11.20 | 0.00 |
| Gestation age |  | 79.95 | 58.01 | 101.88 | <.0001 |  | 79.87 | 57.93 | 101.81 | <.0001 | . | 77.57 | 55.54 | 99.59 | <.0001 |
| African ancestry |  | -116.62 | -355.37 | 122.13 | 0.34 |  | -118.13 | -357.05 | 120.80 | 0.33 |  | -120.07 | -358.59 | 118.45 | 0.32 |

a Model 1a included bGRS, maternal age, education, fasting plasma glucose (mg/dl), hypertension status, baby’s gender, gestational age at delivery, and baby’s proportion of African ancestry;

b Model 2a included Model 1a and mGRS;

c Model 3a included Model 2a and maternal pre-pregnancy BMI

bGRS, Obesity genetic risk score in babies

mGRS, Obesity genetic risk score in mothers

**Table S3. Association of maternal obesity genetic risk with birthweight**

| **Maternal GRS** |  | **Model 1b ^a^** | | | |  | **Model 2b ^b^** | | | |  | **Model 3b ^c^** | | | |
| --- | --- | --- | --- | --- | --- | --- | --- | --- | --- | --- | --- | --- | --- | --- | --- |
|  |  | ***β*** | **(95% CI)** | | ***P*** |  | ***β*** | **(95% CI)** | | ***P*** |  | ***β*** | **(95% CI)** | | ***P*** |
| **Continuous GRS** | | | | | | | | | | | | | | | |
| mGRS |  | 1.15 | -8.91 | 11.21 | 0.82 |  | 7.8 | -3.93 | 19.53 | 0.19 |  | 7.11 | -4.62 | 18.84 | 0.23 |
| bGRS |  |  |  |  |  |  | -12.75 | -24.38 | -1.12 | 0.03 |  | -12.37 | -23.99 | -0.74 | 0.04 |
| ppBMI |  |  |  |  |  |  |  |  |  |  |  | 5.62 | 0.29 | 10.95 | 0.04 |
| Education | Graduate or higher | 164.84 | 1.1 | 328.59 | 0.05 |  | 166.68 | 3.24 | 330.13 | 0.05 |  | 173.37 | 10.08 | 336.65 | 0.04 |
|  | No Graduate Degree | -1.34 | -90.65 | 87.98 | 0.98 |  | 2.58 | -86.66 | 91.82 | 0.95 |  | 3.67 | -85.66 | 93.01 | 0.94 |
|  | High school | -55.64 | -148 | 36.71 | 0.24 |  | -57.21 | -149.41 | 35 | 0.22 |  | -56.51 | -148.68 | 35.65 | 0.23 |
|  | Below high school | *ref* | *ref* | *ref* | *ref* |  | *ref* | *ref* | *ref* | *ref* |  | *ref* | *ref* | *ref* | *ref* |
| Hypertension | Yes | -45.32 | -146.24 | 55.6 | 0.38 |  | -38.63 | -139.54 | 62.28 | 0.45 |  | -63.6 | -167.15 | 39.96 | 0.23 |
|  | No | *ref* | *ref* | *ref* | *ref* |  | *ref* | *ref* | *ref* | *ref* |  | *ref* | *ref* | *ref* | *ref* |
| Gender | Female | -133.95 | -188.71 | -79.2 | <.0001 |  | -132.51 | -187.18 | -77.85 | <.0001 |  | -132.91 | -187.5 | -78.32 | <.0001 |
|  | Male | *ref* | *ref* | *ref* | *ref* |  | *ref* | *ref* | *ref* | *ref* |  | *ref* | *ref* | *ref* | *ref* |
| Age |  | 6.71 | 1.72 | 11.69 | 0.01 |  | 6.69 | 1.72 | 11.66 | 0.01 |  | 5.91 | 0.89 | 10.93 | 0.02 |
| Fasting glucose |  | 8.07 | 4.42 | 11.71 | <.0001 |  | 8.11 | 4.47 | 11.74 | <.0001 |  | 6.96 | 3.17 | 10.75 | 0.00 |
| Gestation age |  | 79.52 | 57.53 | 101.51 | <.0001 |  | 79.1 | 57.15 | 101.05 | <.0001 |  | 76.87 | 54.84 | 98.89 | <.0001 |
| African ancestry |  | -268.31 | -512.35 | -24.27 | 0.03 |  | -262.96 | -506.59 | -19.34 | 0.03 |  | -261.99 | -505.18 | -18.81 | 0.03 |
|  |  |  |  |  |  |  |  |  |  |  |  |  |  |  |  |
| **Categorical GRS** | | | | | | | | | | | | | | | |
| mGRS (high vs low) |  | -9.09 | -63.71 | 45.53 | 0.74 |  | 13.83 | -43.58 | 71.23 | 0.64 |  | 12.17 | -45.16 | 69.5 | 0.68 |
| bGRS (high vs low) |  |  |  |  |  |  | -72.67 | -130.09 | -15.24 | 0.01 |  | -70.09 | -127.49 | -12.69 | 0.02 |
| ppBMI |  |  |  |  |  |  |  |  |  |  |  | 5.55 | 0.22 | 10.88 | 0.04 |
| Education | Graduate or higher | 163.97 | 0.28 | 327.66 | 0.05 |  | 163.28 | 0.05 | 326.52 | 0.05 |  | 170.16 | 7.07 | 333.25 | 0.04 |
|  | No Graduate Degree | -1.16 | -90.48 | 88.15 | 0.98 |  | -1.79 | -90.84 | 87.25 | 0.97 |  | -0.62 | -89.76 | 88.51 | 0.99 |
|  | High school | -56.12 | -148.5 | 36.26 | 0.23 |  | -60.48 | -152.65 | 31.7 | 0.20 |  | -59.66 | -151.78 | 32.47 | 0.20 |
|  | Below high school | *ref* | *ref* | *ref* | *ref* |  | *ref* | *ref* | *ref* | *ref* |  | *ref* | *ref* | *ref* | *ref* |
| Hypertension | Yes | -46.13 | -147.1 | 54.84 | 0.37 |  | -43.06 | -143.79 | 57.67 | 0.40 |  | -67.6 | -170.94 | 35.74 | 0.20 |
|  | No | *ref* | *ref* | *ref* | *ref* |  | *ref* | *ref* | *ref* | *ref* |  | *ref* | *ref* | *ref* | *ref* |
| Gender | Female | -133.63 | -188.4 | -78.86 | <.0001 |  | -134.16 | -188.78 | -79.54 | <.0001 |  | -134.5 | -189.05 | -79.96 | <.0001 |
|  | Male | *ref* | *ref* | *ref* | *ref* |  | *ref* | *ref* | *ref* | *ref* |  | *ref* | *ref* | *ref* | *ref* |
| Age |  | 6.79 | 1.81 | 11.77 | 0.01 |  | 6.79 | 1.82 | 11.76 | 0.01 |  | 6.01 | 0.99 | 11.03 | 0.02 |
| Fasting glucose |  | 8.09 | 4.45 | 11.73 | <.0001 |  | 8.3 | 4.66 | 11.94 | <.0001 |  | 7.16 | 3.36 | 10.95 | 0.00 |
| Gestation age |  | 79.72 | 57.76 | 101.67 | <.0001 |  | 79.8 | 57.91 | 101.7 | <.0001 |  | 77.52 | 55.55 | 99.5 | <.0001 |
| African ancestry |  | -269.1 | -513 | -25.19 | 0.03 |  | -271.17 | -514.41 | -27.93 | 0.03 |  | -269.58 | -512.41 | -26.75 | 0.03 |

^a^ Model 1b included mGRS, maternal age, education, fasting plasma glucose (mg/dl), hypertension status, baby’s gender, gestational age at delivery, and mother’s proportion of African ancestry;

^b^ Model 2b included Model 1b + bGRS;

^c^ Model 3b included Model 2b + maternal pre-pregnancy BMI

bGRS, Obesity genetic risk score in babies

mGRS, Obesity genetic risk score in mothers

**Supplementary Figure 1.** Retrospective power analysis for the interaction analysis on the full model using standard deviation of 150 gm for 950 mother-baby pairs.

1. Interaction between mother prepregnancy BMI and mother genetic risk score adjusting for all the study covariates were as shown in the figure ((mGRS*mBMI power)

Model Birthweight = mGRS, maternal age, education, fasting plasma glucose, hypertension status, baby’s sex, gestational age, the proportion of mother’s African ancestry + bGRS + mBMI+ mGRS x mBMI


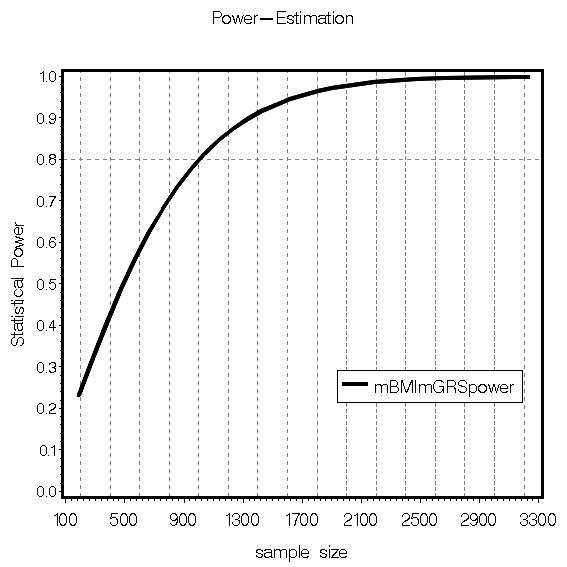


1. Interaction between baby and mother genetic risk score adjusting for all the study covariates were as shown in the figure (mGRS*bGRS power)

Model Birthweight = mGRS, maternal age, education, fasting plasma glucose, hypertension status, baby’s sex, gestational age, the proportion of mother’s African ancestry + bGRS + mGRS x bGRS


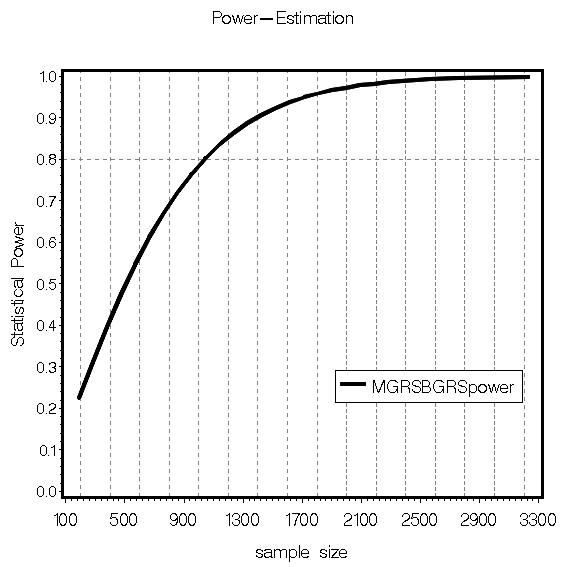

Supplement: Supplementary file 1 [file Table_1.DOCX]
